# Prevalence and Antibiotic Resistance against Tetracycline in *Campylobacter jejuni* and *C. coli* in Cattle and Beef Meat from Selangor, Malaysia

**DOI:** 10.3389/fmicb.2017.02254

**Published:** 2017-12-04

**Authors:** Jayasekara M. K. J. K. Premarathne, Aimi S. Anuar, Tze Young Thung, Dilan A. Satharasinghe, Nuzul Noorahya Jambari, Noor-Azira Abdul-Mutalib, John Tang Yew Huat, Dayang F. Basri, Yaya Rukayadi, Yoshitsugu Nakaguchi, Mitsuaki Nishibuchi, Son Radu

**Affiliations:** ^1^Faculty of Food Science and Technology, Food Safety Research Center, Universiti Putra Malaysia, Seri Kembangan, Malaysia; ^2^Department of Livestock and Avian Science, Faculty of Livestock, Fisheries and Nutrition, Wayamba University of Sri Lanka, Kuliyapitiya, Sri Lanka; ^3^Institute of Bio Science, Universiti Putra Malaysia, Seri Kembangan, Malaysia; ^4^Department of Basic Veterinary Science, Faculty of Veterinary Medicine and Animal Science, University of Peradeniya, Peradeniya, Sri Lanka; ^5^Food Safety Research Center (FOSREC), Faculty of Food Science and Technology, Universiti Putra Malaysia, Seri Kembangan, Malaysia; ^6^Faculty of Food Technology, Universiti Sultan Zainal Abidin, Kuala Terengganu, Malaysia; ^7^Faculty of Health Sciences, School of Diagnostic and Applied Health Sciences, Universiti Kebangsaan Malaysia, Kuala Lumpur, Malaysia; ^8^Center for Southeast Asian Studies, Kyoto University, Kyoto, Japan; ^9^Laboratory of Food Safety and Food Integrity, Institute of Tropical Agriculture and Food Security, Universiti Putra Malaysia, Seri Kembangan, Malaysia

**Keywords:** *Campylobacter*, prevalence, beef, antibiotic suspetibility, MPN-PCR

## Abstract

*Campylobacter* is a major foodborne pathogen frequently associated with human bacterial gastroenteritis in the world. This study was conducted to determine the prevalence and antibiotic resistance of *Campylobacter* spp. in the beef food system in Malaysia. A total of 340 samples consisting of cattle feces (*n* = 100), beef (*n* = 120) from wet markets and beef (*n* = 120) from hypermarkets were analyzed for *Campylobacter* spp. The overall prevalence of *Campylobacter* was 17.4%, consisting of 33% in cattle fecal samples, 14.2% in raw beef from wet market and 7.5% in raw beef from the hypermarket. The multiplex-polymerase chain reaction (PCR) identified 55% of the strains as *C. jejuni*, 26% as *C. coli*, and 19% as other *Campylobacter* spp. A high percentage of *Campylobacter* spp. were resistant to tetracycline (76.9%) and ampicillin (69.2%), whilst low resistance was exhibited to chloramphenicol (7.6%). The MAR Index of *Campylobacter* isolates from this study ranged from 0.09 to 0.73. The present study indicates the potential public health risk associated with the beef food system, hence stringent surveillance, regulatory measures, and appropriate interventions are required to minimize *Campylobacter* contamination and prudent antibiotic usage that can ensure consumer safety.

## Introduction

Thermophilic *Campylobacter* is a major bacterial pathogen that causes foodborne infections around the world (EFSA, 2011; WHO, 2012). *C. jejuni* and *C. coli* have been identified as the most common species that lead to campylobacteriosis (Silva et al., 2011). The high incidence rate of campylobacteriosis can be associated with the low minimal infective dose of thermophilic *Campylobacter* which is around 500–800 cells (Nachamkin et al., 2008). *Campylobacter* spp. colonize the enteric tract of birds, sheep, cattle and pigs (Stanley and Jones, 2003; Humphrey et al., 2007). Food originated from animals act as the primary source of *Campylobacter* infection (Doorduyn et al., 2010).

Reduce susceptibility of foodborne pathogens to antimicrobials significantly affect global public health (Chatre et al., 2010; Aarestrup, 2015). Increasing resistance in *Campylobacter* to antimicrobials particularly to tetracycline, erythromycin, and (fluoro)quinolones (Gaudreau and Gilbert, 2003; Zhu et al., 2006) was associated with reduce response to therapy leading to higher morbidity and mortality rates in humans (Zhu et al., 2006).

*Campylobacter* a major public health concern around the world, especially in developed countries and these countries have *Campylobacter* surveillance systems (Scallan et al., 2011; EFSA and ECDC, 2014). Even in the South-East Asia, *Campylobacter* is becoming a key foodborne pathogen (Premarathne et al., 2017). According to a hospital-based study conducted by Lee and Puthucheary (2002), *Campylobacter* spp. was reported at 5% prevalence level and placed among the first five causative agents associated with child's diarrhea. Furthermore, *Campylobacter* were reported in humans from Indonesia (Tjaniadi et al., 2003), Lao People's Democratic Republic (Yamashiro et al., 1998), Singapore (Chau et al., 2016), and Thailand (Bodhidatta et al., 2013). Singapore is the only South-East Asian country that performs national surveillance of *Campylobacter* infection in humans. Estimation and control of campylobacteriosis in South-East Asian countries, including Malaysia, is hindered due to insufficient regulations for food safety and inadequate data on epidemiology of *Campylobacter* cases (Premarathne et al., 2017). Therefore, prevalence and antimicrobial resistance of *Campylobacter* are important to be assessed in these countries.

In Malaysia, beef is sold in traditional wet markets and modern hypermarkets (Chamhuri and Batt, 2013). Beef available in wet markets were supplied by locally slaughtered and dressed cattle at the municipal abattoir or none-abattoir premises (Marimuthu et al., 2015). The wet markets were operated for a short time around 6 h per day and were able to provide fresh meat every day (Tang et al., 2009). The hypermarkets offered chilled or frozen meat that locally produced or from imported beef (Ariff et al., 2015). According to a consumer survey, Malaysians considered that fresh meat is more succulent, healthier and assure the halalness. Therefore, they preferred purchasing meat from wet markets over hypermarkets (Chamhuri and Batt, 2013).

Naturally *Campylobacter* are present in cattle; therefore, feces can contaminate the beef carcasses during slaughtering (Hakkinen et al., 2007). Further, a study conducted in Malaysisa found that *C. jejuni* can survive in food processing environments through formation of biofilms (Teh et al., 2014). The prevalence of *Campylobacter* species in poultry (Tang et al., 2009; Mansouri-Najand et al., 2012; Rejab et al., 2012), duck (Nor Faiza et al., 2013) and salad vegetables (Chai et al., 2007) have been reported in Malaysia. However, limited data exist on the prevalence of *Campylobacter* in other food commodities, including beef food system. Impelled by scarcity of data, this study was conducted to determine the prevalence of *Campylobacter* in the beef food system together with assessing the antibiogram profiles of *Campylobacter* spp.

## Materials and methods

### Bacterial strains

In this study *C. jejuni* ATCC 33221 and *C. coli* ATCC 33559 were used as the positive controls.

### Sample collection

A total of 100 fecal samples were collected from apparently healthy beef cattle in three different farms, while purchased 120 chilled beef samples from hypermarkets (*n* = 6) and 120 fresh beef samples from wet markets (*n* = 6) in Selangor, Malaysia. All collected samples were immediately transported to the laboratory in ice and processed within the same day of sample collection.

### Sample processing

The standardized and published protocol by Chai et al. (2007) and Tang et al. (2009) was used for sample processing. A 10 g portion of the sample was aseptically placed in a stomacher bag containing 90 mL Bolton broth base (Merck KGaA, Darmstadt, Germany) and homogenized using a stomacher lab-blender (Interscience, France) at normal speed for 60 s. The Bolton broth was supplemented with Bolton broth selective supplement contained Vancomycin, Cefoperazone, Trimethoprim Lactate and Amphotericin B (Merck KGaA) and 5% lysed horse blood.

Control samples were prepared only with supplemented Bolton broth and inoculated with *C. jejuni* ATCC 33221 and *C. coli* ATCC 33559. The controls and samples were incubated at 42°C for 48 h under microaerophilic conditions comprised of 8–10% (v/v) CO_2_ and 5–6% (v/v) O_2_ generated by the Anaerocult C system (Merck KGaA) in an anaerobic jar.

### Microbiological isolation

After incubation, a loop full of the enriched sample was streaked in duplicates onto modified charcoal-cefoperazone-deoxycholate agar (mCCDA; Merck KGaA) with CCDA selective supplement contained Cefoperazone and Amphotericin B (Merck KGaA) and incubated microaerobically (Anaerocult C system; Merck KGaA) for 48 h at 42°C. Presumptive identification of *Campylobacter* colonies was based on ISO method that includes (ISO, 2006a,b); oxidase test, catalase test, gram-staining and typical microscopic *Campylobacter* morphology. The presumptive colonies were confirmed by using PCR (Polymerase Chain Reaction) assay.

### MPN-enrichment

*Campylobacter* cells were counted using the MPN method with triplicate of three 10-fold dilution series prepared by transferring 100 μL of homogenized sample into 900 μL of Bolton broth base (Merck KGaA) supplemented with Bolton broth selective supplements. A control tube was prepared with supplemented Bolton broth and inoculated with *C. jejuni* ATCC 33221 and *C. coli* ATCC 33559. The MPN tubes were incubated at 42°C for 48 h under microaerophilic conditions generated by the Anaerocult C system (Merck KGaA) in an anaerobic jar.

### DNA extraction

Bacterial DNA was extracted from the enriched sample tubes and presumptive bacterial colonies using boiled-cell method (Tang et al., 2009). Briefly, enriched samples in the tubes were pelleted by centrifuging at 10,000 × g for 10 min. The supernatant was carefully removed, next the pellet was washed once with 500 μL sterile distilled water and harvested bacterial cells were suspended in 500 μL of sterile TE buffer (pH 8.0). Bacterial cells were lysed by subjecting to boiling for 10 min, followed by rapid cooling at −20°C for 10 min. Next the sample was centrifuged at 10,000 × g for 10 min. Finally, the supernatant containing bacterial DNA was stored at −20°C until used in PCR.

### PCR identification

All MPN tubes were subjected to identification of *Campylobacter* spp. by multiplex PCR reaction using primers specific for *Campylobacter* genes, *C. jejuni* and *C. coli* (Table 1).

**Table 1:** PCR primers set used for detection of *Campylobacter* spp., *C. jejuni* and *C. coli*.

**Targeted species**	**Targeted gene**	**Sequence 5′-3′**	**Amplicon size (bp)**	**References**
*Campylobacter*	*Cadf*	F:TTG AAG GTA ATT TAG ATA TG	400	Konkel et al., 1999
		R:CTA ATA CCT AAA GTT GAA AC		
*C. jejuni*	*Oxidoreductase subunit*	F:ATG AAA AAA TAT TTA GTT TTT GCA	160	Winters and Slavik, 1995
		R:ATT TTA TTA TTT GTA GCA GCG		
*C. coli*	*Ceu*	F:CAA ATA AAG TTA GAG GTA GAA TGT	894	Gonzalez et al., 1997
		R:GGA TAA GCA CTA GCT AGC TGA T		

The PCR was carried out in 25 μL reactions, and each reaction contained 5 μL of 5 × PCR buffer (Promega, USA); 3 μL of 25 mM MgCl_2_ (Promega, USA); 1 U *Taq* Polymerase (Promega, USA); 1 μL of 10 mM deoxynucleoside triphosphate (dNTP) mix (Promega, USA); 1 μM of forward and reverse primer (Sigma, UK) and 5 μL of template DNA.

The cycling conditions were set as follows on the VeritiTM 96-Well Thermal Cycler (Applied Biosystems, USA), initial denaturing at 94°C for 4 min followed by 33 cycles of denaturing at 94°C for 1 min, annealing at 50°C for 1 min and extension at 72°C for 1 min followed by final extension at 72°C for 5 min.

A 5 μL of all PCR amplicons were horizontally electrophoresed through a 1.25% agarose gel stained with ethidium bromide in 1 × Tris-acetate-EDTA (TAE) (1 mM EDTA, 40 mM Tris-acetate) buffer at 90 V for 40 min. The agarose gel was visualized under ultraviolet (UV) light transilluminator (SynGene, Frederick, USA) and photographed. In each gel a positive control, non-template control and a DNA-molecular marker (100-bp ladder) (Vivantis Technologies, Malaysia) were included. The DNA extracted from *C. coli* (ATCC 33559) and *C. jejuni* (ATCC 33560) were used as the positive controls, while PCR mixture without DNA template was used as the non-template control.

### Antibiotic susceptibility testing

Antimicrobial susceptibility tests were conducted using following antimicrobial impregnated disks (Oxoid, England, UK); ampicillin (AMP; 10 μg), cephalothin (CEF; 30 μg), chloramphenicol (CHL; 30 μg), ciprofloxacin (CIP; 5 μg), enrofloxacin (ENR; 5 μg), erythromycin (ERY; 15 μg), gentamicin (GEN; 10 μg), norfloxacin (NORr; 10 μg), nalidixic acid (NAL; 30 μg), sterptomycin (STR; 25 μg), and tetracycline (TET; 30 μg) according to the standard Kirby- Bauer disk diffusion method (Bauer et al., 1966) and performed according to the recommendations of the Clinical Laboratory Standards Institute (Clinical and Laboratory Standards Institute, 2012).

All isolates were revived from glycerol stocks using Bolton broth supplemented with 5% lysed horse blood. The suspensions were incubated for 48 h at 42°C under microaerophilic conditions. Revived cultures were grown in Brain heart infusion (BHI: Merck KGaA) broth at 42°C under microaerophilic conditions for 24 h and the turbidity of the suspension was adjusted to 0.5 McFarland standard (Clinical and Laboratory Standards Institute, 2012). Then the culture was swabbed uniformly using sterile cotton swabs onto MH agar plates (Merck KGaA) supplemented with 5% horse blood. The plates were incubated under microaerophilic conditions (Anaerocult C system; Merck KGaA) at 37°C for 48 h. *C. jejuni* ATCC 33560 and *C. coli* ATCC 33559 were used as reference strains.

The diameters of the inhibition zones around the antibiotic disk were measured. The breakpoints used to categorize isolates as susceptible (s), intermediate (i) and resistant (r) were based on CLSI recommendations (Clinical and Laboratory Standards Institute, 2012). As there were no CLSI recommendations for *Campylobacter* strains, the standards assigned to the *Enterobacteriaceae* family were used as breakpoints to interpret *Campylobacter* resistance.

### Multiple antimicrobial resistance (MAR) indexing

Multi-resistance of *Campylobacter* isolates were quantified using the MAR indexing.

$$\begin{array}{l} \text{MAR index }=\text{ a}/\text{b} \end{array}$$

where “a” indicate the number of antimicrobials to which the particular isolate was resistant and “b” indicate the total number of antimicrobials to which the particular isolate was tested (Krumperman, 1983).

### Statistical analysis

The difference in prevalence level between the two detection methods (MPN_plating and MPN_PCR) and various sample types obtained from cattle, wet market and hypermarket were analyzed using Pearson chi-square test (X^2^ test). The results were considered statistically significant at *P* < 0.05 at 95% confidence level.

## Results

### Culture-based detection and colony confirmation applying PCR method

Presumptive *Campylobacter* spp. were detected in 8% (8 of 100 samples) of the cattle feces samples and 1.6% (4 of 240) of the beef meat samples and all presumptive isolates were confirmed as *Campylobacter* spp. by PCR.

### MPN-plating media based enumeration

The prevalence of *Campylobacter* spp. in cattle and beef meat samples determined by using the MPN-plating is presented in Table 2. The MPN-plating detected *Campylobacter* spp. in 3.5% of the total samples (Table 2). *Campylobacter* was detected only in 8 out of 100 fecal samples, including *C. jejuni* in 5% and *C. coli* in 2% of the fecal samples. The prevalence of *C. jejuni* was significantly higher (*P* ≤ 0.05) than the *C. coli* in fecal samples.

**Table 2:** Culture-based detection of *Campylobacter* spp. in cattle and beef.

**Source**	**Sample type**	**Sample size**	***C. jejuni***	***C. coli***	***C. jejuni/C. coli***	**OTC**	**Total**
Beef cattle	Feces	100	16 (16)	5 (5)	8 (8)	4 (4)	33 (33)
Wet market	Beef	120	8 (6.7)	1 (0.8)	3 (2.5)	5 (4.2)	17 (14.2)
Hyper market	Beef	120	6 (5)	2 (1.7)	0 (0)	0 (0)	9 (7.5)
	Total	340	30 (8.8)	8 (6.7)	11 (9.2)	10 (8.2)	59 (17.4)

*OTC, Other thermophilic Campylobacter spp*.

The majority (66%) of isolates were identified as *C. jejuni* and the remainder as *C. coli* (34%). *Campylobacter* spp. were detected only in 2% of the beef samples from wet and hypermarket by the MPN-plating method. The MPN-plating found *C. jejuni* and *C. coli* in 0.8% of beef samples from wet and hypermarket (Table 2). The prevalence of *Campylobacter* spp. was statistically not significant (*P* ≤ 0.05) between the wet and hypermarkets. However, the prevalence of *Campylobacter* spp. in fecal samples was significantly higher (*P* ≤ 0.05) than that in beef samples.

### MPN-PCR-based enumeration

The prevalence of *Campylobacter* spp determined by using the MPN-PCR method is presented in Table 3. Based on the MPN-PCR results *Campylobacter* spp. was detected in 59 (17.4%) of the total 340 examined samples. Detection of *Campylobacter* spp. by MPN-PCR method was significantly higher (*P* ≤ 0.05) comparative to the MPN-plating (Table 3).

**Table 3:** MPN-PCR based detection of *Campylobacter* spp. in cattle and beef.

**Source**	**Sample type**	**Sample size**	***C. jejuni***	***C. coli***	***C. jejuni/C. coli***	**OTC**	**Total**
Beef cattle	Feces	100	16 (16)	5 (5)	8 (8)	4 (4)	33 (33)
Wet market	Beef	120	8 (6.7)	1 (0.8)	3 (2.5)	5 (4.2)	17 (14.2)
Hyper market	Beef	120	6 (5)	2 (1.7)	0 (0)	0 (0)	9 (7.5)
	Total	340	30 (8.8)	8 (6.7)	11 (9.2)	10 (8.2)	59 (17.4)

*OTC, Other thermophilic Campylobacter spp*.

The MPN-PCR method detected *Campylobacter* in 33 out of 100 cattle fecal samples. According to the MPN-PCR, fecal samples contained 16, 8, and 5% of *C. jejuni*, both *Campylobacter* spp. and *C. coli* respectively. The prevalence of *C. jejuni* was significantly higher (*P* ≤ 0.05) than the *C. coli* in fecal samples.

*Campylobacter* was detected in a total of 17/120 (14.2%) beef from wet markets and in 9/120 (7.5%) beef from hypermarkets (Table 3). The predominant species of *Campylobacter* detected in beef samples from wet market (55%) was *C. jejuni* and remainder was *C. coli* (20%) and other *Campylobacter* spp. (25%). Similarly, at the hypermarket, *C. jejuni* was the most prevalent (67%) while *C. coli* and other *Campylobacter* spp. were detected in 22 and 11% of the beef samples respectively. The prevalence of *Campylobacter* spp. was statistically not significant (*P* ≤ 0.05) between the wet and hypermarkets.

*Campylobacter* concentration in cattle and beef was enumerated using the MPN method (Table 4). The highest number of *Campylobacter* found in fecal samples from cattle, including 3-460 MPN/g of *C. jejuni* and 3-43 MPN/g of *C. coli* and 3-75 MPN/g of other thermophilic *Campylobacter* spp. Beef from wet markets observed to carry *Campylobacter* spp. in the range of 3-75 MPN/g while the beef samples from hypermarkets harbored low *Campylobacter* spp. concentration ranged from 3 to 15 MPN/g.

**Table 4:** Concentration of *Campylobacter* spp. in cattle and beef.

**Source**	**Sample type**	**Positive sample no**	***C. jejuni***			***C. coli***			**OTC**		
			**Max**	**Med**	**Min**	**Max**	**Med**	**Min**	**Max**	**Med**	**Min**
Farm	Feces	33	460	6.2	3	43	6.1	3	75	15	3
Wet market	Beef	17	29	3.6	3	3.6	3	3	75	21	3
Hyper market	Beef	9	6.1	3	3	15	9.3	3.6	ND	ND	ND

*Max, Maximum; Med, Median; Min, Minimum; OTC, Other thermophilic Campylobacter spp., ND, not detected*.

### Antimicrobial resistance testing

Eleven antimicrobials named under the different antimicrobial groups were employed in this study to determine the resistance of *Campylobacter* isolates. All the isolates were resistant at least one or more antimicrobials (Table 5). Antimicrobial resistance pattern among the tested *Campylobacter* spp. Indicated that majority of the isolates were resistant to tetracycline (76.9%) and ampicillin (69.2%). Least resistance was observed for chloramphenicol (7.6%), cephalothin (15.4%), ciprofloxacin (15.4%) and gentamicin (15.4%).

**Table 5:** The frequency of antibiotic drug resistance in *Campylobacter* spp.

**Antibiotic group**	**Resistance profile**	**Resistant isolates**		
		**No %**	***C. jejuni***	***C. coli***
Aminoglycosides	Gentamycin	2 (15.4)	1	1
	Streptomycin	3 (23.1)	1	2
Macrolides	Erythromycin	4 (30.8)	1	3
Quinolones and Fluoroquinolones	Ciprofloxacin	2 (15.4)	2	0
	Enrofloaxacin	5 (38.5)	2	3
	Nalidixix acid	7 (53.8)	4	3
	Norfloaxacin	4 (30.8)	2	2
Tetracyclines	Tetracycline	10 (76.9)	5	5
Chloramphenicol	Chloramphenicol	1 (7.6)	0	1
Penicillins	Ampicillin	9 (69.2)	5	4
Cephalexins	Cephalothin	2 (15.4)	0	2

The MAR index of *Campylobacter* species that were isolated from the current study indicate in Table 6. *Campylobacter* species exhibited 7 different antibiotic resistant patterns with MAR index ranging from 0.09 to 0.73. The highest MAR index was 0.73 and showed S, E, En, Na, Nor, Te, C, Amp resistant pattern. Meanwhile, lowest MAR index of 0.09 was demonstrated in isolates that indicate resistance to Te or Amp (Table 5). Moreover, 53.8% of the isolates were resistant to the three or more antimicrobials and demonstrated the Multi Drug Resistance (MDR).

**Table 6:** Multiple antibiotic resistance (MAR) index of *Campylobacter* spp.

**MAR index**	***Campylobacter* spp**.	**Isolate code**	**Antibiogram**
0.09	*C. jejuni*	CF3	TET
0.09	*C. coli*	BH158	TET
0.09	*C. jejuni*	CF58	TET
0.09	*C. jejuni*	CF85	AMP
0.18	*C. jejuni*	CF47	NAL, AMP
0.18	*C. jejuni*	BH70	TET, AMP
0.27	*C. jejuni*	BW188	NAL, TET, AMP
0.36	*C. coli*	CF46	NAL, TET, AMP, CEF
0.55	*C. coli*	BW189	STR, ERY, ENR, TET, AMP, CEF
0.55	*C. jejuni*	CF70	NAL, CIP, ENR, NOR, TET, AMP
0.64	*C. coli*	CF84	GEN, ERY, ENR, NAL, NOR, TET, AMP
0.64	*C. jejuni*	CF84	GEN, STR, ERY, CIP, ENR, NAL, NOR
0.73	*C. coli*	CF69	STR, ERY, ENR, NAL, NOR, TET, CHL, AMP

## Discussion

Though the prevalence of *Campylobacter* in chicken and broilers was well documented in Malaysia; no or limited information was available for *Campylobacter* in other animals and food commodities, particularly for cattle and beef in the country. Therefore, the present study aimed to address the prevalence, concentration and antimicrobial resistant profiles of thermophilic *Campylobacter* species present in cattle fecal samples and beef.

The overall prevalence of thermophilic *Campylobacter* spp. in fecal samples of apparently healthy cattle was 33% (Table 2). Findings of this study was similar to the previous findings reported on prevalence level of *Campylobacter* spp. in cattle from countries including; Japan (39.6%) (Haruna et al., 2013) and Chile (35.9%) (Fernández and Hitschfeld, 2009). Sanad et al. (2011) found only 19.2% prevalence level in samples collected from feedlot, mature cows and bulls presented for slaughter across USA. Meanwhile, low mean *Campylobacter* prevalence level of 5.6% (Nonga et al., 2010) and 13.2% (Karikari et al., 2017) was reported from Tanzania and Ghana respectively. However, a study conducted in 5 different states in the U.S. reported a high prevalence (72.2%) of *Campylobacter* in feedlot cattle (Tang et al., 2017). While, 75–83.3% *Campylobacter* prevalence level was reported in dairy cattle from Lithuania (Ramonaite et al., 2013). Therefore, this study also contributes to prior discussions that cattle can act as a potential reservoir for transmitting *Campylobacter* spp. into the food system (Karenlampi et al., 2007; Cha et al., 2017). In the present study, *C. jejuni* (58.5%) was the predominantly isolated organism in cattle followed by *C. coli* (31.7%), and other thermophilic *Campylobacter* spp. The prevalence of *C. jejuni* and *C. coli* detected in this study was consistent with previous studies (Haruna et al., 2013). However, Karikari et al. (2017) reported higher prevalence level of *C. coli* (47.8%) comparatively *C. jejuni* was detected in 35.8% samples. Similarly, *C. jejuni* was detected in 35.2% cattle feacal samples with a high prevelance of *C. coli* (72.9%) (Sanad et al., 2011). However, Okunlade et al. (2015) reported low *C. coli* prevalence (20.4%) level in rectal swabs collected from cattle from Ibadan, Oyo State, Nigeria. A study conducted in Michigan, USA reported 69.2% prevalence level for *C. jejuni* (Cha et al., 2017). Other *Campylobacter* spp. (0.09%) detected in this study needs to be further characterized. Differences in the reported prevalence level can be resultant due to geographical location, sample size and method of analysis.

The low contamination frequency of *Campylobacter* species detected in beef in this study was in agreement with recent studies. The *Campylobacter* prevalence of fresh beef samples from Poland was 10.1% in Korsak et al. (2015), 16.2% in retail ground beef from Saskatchewan, Canada (Trokhymchuk et al., 2014) and 17.4% in whole beef cuts from retail shops in USA (Vipham et al., 2012). Meanwhile, some studies conducted previously could only detect very low *Campylobacter* contamination level in beef, including 1.9% in Tanzania (Nonga et al., 2010) and 3.3% in Belgium (Ghafir et al., 2007). Chilling reduce the surface humidity of red meat which can be related to low prevalence level *Campylobacter* in beef compared to chicken as *Campylobacter* is very sensitive to dehydration (Silva et al., 2011). The current study detected high *Campylobacter* prevalence in beef from wet markets (14.2%) comparative to beef from hypermarkets (7.5%) can be associated with the availability of fresh meat in the wet markets in Malaysia. Fresh meat is sold without chilling in the wet markets as it is considered fresh and succulent since it retains moisture. This may facilitate the survival of *Campylobacter* on raw red meat. Further, the low hygienic conditions in the wet markets (Chamhuri and Batt, 2013) may also contribute to the comparatively higher *Campylobacter* contamination rate and increase the potential for cross contamination. Moreover, no-abattoir slaughtering procedures (Marimuthu et al., 2015) can also result in high contamination of beef in wet markets with *Campylobacter*. We speculate that the main reason for the difference of *Campylobacter* prevalence in cattle and beef from various countries can be the result of differences in sampling methods, storage duration, microbiological and molecular methods employed. The results of this study indicate that not only cattle but also beef can be a potential reservoir for *Campylobacter* infection.

Findings of this study reported that *Campylobacter* spp. in cattle range from 460-3 MPN/g, while a lower concentration was detected in beef (29-3 MPN/g). Further this study was in line with Nielsen (2002) who reported that mean *Campylobacter* concentration in cattle feces was 126 MPN/g. A study conducted by Stanley et al. (1998) observed a maximum of *Campylobacter* concentration at the level of 6.3 × 10^7^ MPN/g in cattle. *Campylobacter* concentration in cattle intestine can be lower than that of broilers (Stanley and Jones, 2003). However, owed to a very low infective dose of *Campylobacter* the reported prevalence and concentration indicate the potential of contaminating beef during slaughter procedure. Therefore, proper sanitary and food safety measures should be implemented to ensure safety of beef. Further, an upward trend was observed among Malaysian consumers toward higher value meat such as beef and mutton (Yeong-Sheng et al., 2008). Therefore, beef can be a potential source of *Campylobacter* infection and need more attention focused on improving safety.

Isolation and identification of *Campylobacter* using conventional culture based methods can be challenging due to the fastidious growth requirements and discrepancies in biochemical tests (Nachamkin et al., 2008). Application of molecular methods for identification of foodborne pathogens has increasingly used for food samples to complement conventional microbiological methods owing to its rapid turnaround time and sensitivity (Inglis and Kalischuk, 2003). In this study, detection of *Campylobacter* spp. using PCR method was higher than plating on mCCDA agar. Previous studies on the prevalence of foodborne pathogens in food indicate a difference between the findings through the molecular biological methods comparative to findings of culturing methods (Inglis and Kalischuk, 2003; Chai et al., 2007; Tang et al., 2009). When growth conditions are not favorable *Campylobacter* cells can transition from vegetative stage into a viable but non-culturable (VBNC) state, in which the bacteria cannot be cultured using conventional culture methods (Oliver, 2010; Ramamurthy et al., 2014). The molecular amplification techniques can overcome the limitation of detecting VBNC cells with providing high specificity and sensitivity (Singh et al., 2011).

The high resistance detected in *Campylobacter* isolates against tetracycline in the current study was consistent with previously reported work. (Hong et al., 2007; Kashoma et al., 2015). Hong et al. (2007) reported that 93.4% *Campylobacter* strains from Korean beef samples were resistant to multiple antimicrobials and detected high resistance to tetracycline (94.6%). *Campylobacter* isolated from raw beef in Iran demonstrated the highest resistance to tetracycline (Rahimi et al., 2013). *Campylobacter* resistance to the tetracycline was mainly mediated through *tet* (O) plasmid (Pratt and Korolik, 2005) and worldwide 60–100% *C. jejuni* and *C. coli* were reported to carry tetracycline-resistant plasmids (Lee et al., 1994; Kim et al., 2010). High resistance to tetracycline in this study may be resultant due to *tet* (O) plasmid; however *Campylobacter* isolates from Malaysia need further investigation on prevalence and resistance profile of *tet* (O) plasmid. Similar to findings from the current study, the majority of the *Campylobacter* strains from food animals in Tanzania were resistant to ampicillin (70.3%) (Kashoma et al., 2015). Inherently resistance can be overserved in *Campylobacter* strains to β-lactams including ampicillin (Engberg, 2006; Li et al., 2007) which may be contributory to high ampicillin resistance observed in this study. The high resistance to tetracycline and ampicillin detected in this study could be associated with frequent usage of those antimicrobials in humans and animal husbandry (Chopra and Roberts, 2001; Hao et al., 2014).

Least resistance was observed for chloramphenicol, cephalothin, ciprofloxacin and gentamicin. Similarly low resistance was detected against gentamicin (1.8%) and chloramphenicol (4.5%) in the *Campylobacter* isolates from Tanzania (Kashoma et al., 2015). Further, *Campylobacter* spp. isolated from raw meat in Iran were resistant to gentamicin and chloramphenicol 3.2 and 6.5%, respectively (Rahimi et al., 2013). Contrary to the current study, *Campylobacter* isolates in beef from Korea showed high resistant to ciprofloxacin (95.9%) and nalidixic acid (94.6%) in Hong et al. (2007). Discrepancies and similarities in antibiotic resistance patterns can be attributed to variation in sample type, sampling procedure, type and frequency of antibiotic usage in animal husbandry practices and human therapy.

In the current study multiple antibiotic resistances was detected only in 53.8% of the isolates. Slightly higher multiple antibiotic resistance was observed in *Campylobacter* isolates from broilers in Malaysia (Saleha, 2002) and beef liver from USA (Noormohamed and Fakhr, 2014). The present study, 46.2% *Campylobacter* isolates reported to have a MAR less than 0.2. A bacterium that has a MAR index less than 0.2 has been identified to be isolated from animals that antimicrobials were seldom used. While, if the strain has a MAR index greater than 0.2 considered to be originated from producing animals that have a high potential for contamination (Marian et al., 2012). According to the National Pharmaceutical Control Bureau (NPCB) of the Ministry of Health, Malaysia, more than 97 antimicrobials have been registered for use in producing animals in Malaysia. However, comparative to poultry, a lower number of products has been registered to be used in cattle. The MAR index detected in *Campylobacter* isolates from cattle and beef of this study may be associated with low frequency of antibiotic usage in cattle compared to poultry in Malaysia. However, multiple antibiotic resistances associated with isolates from cattle and beef exacerbate the public health concern associated with *Campylobacter* infections.

## Conclusion

Based on the findings of this study indicate the importance of considering the potential public health risk associated with *Campylobacter* in the beef food system in Malaysia. Therefore, implementation of good hygienic practices at the farm, slaughterhouse, and retail level can minimize the *Campylobacter* contamination. Furthermore, the presence of multiple antibiotic resistant *Campylobacter* spp. urge the prudent use of antimicrobials in animal husbandry, farmer awareness and application of good veterinary practices to minimize the likelihood of emerging superbugs.

## Author contributions

JP and AA conducted the experiment. TT and DS did the data analysis. JP prepared the manuscript. NJ, N-AA-M, JH, DB, YR, YN, MN, and SR supervised and assisted in the preparation of the manuscript. NJ and N-AA-M provided consumables.

### Conflict of interest statement

The authors declare that the research was conducted in the absence of any commercial or financial relationships that could be construed as a potential conflict of interest.
